# Dentoalveolar compensation in different anterioposterior and vertical skeletal malocclusions 

**DOI:** 10.4317/jced.56020

**Published:** 2019-08-01

**Authors:** Maged-Sultan Alhammadi

**Affiliations:** 1 Assistant Professor, Division of Orthodontics and Dentofacial Orthopedics, Department of Preventive Dental Sciences , College of Dentistry, Jazan University, Jazan, Saudi Arabia

## Abstract

**Background:**

To evaluate the position and inclination of maxillary and mandibular incisors in adults with different anteroposterior and vertical skeletal malocclusions.

**Material and Methods:**

In this retrospective study, lateral cephalometry of 272 adults (134 males and 138 females) who met the selection criteria were digitally analyzed. They were classified based on both linear and angular measurements, anterioposteriorly into skeletal Class I, Class II and Class III and vertically into hypodivergent, normodivergent and hyperdivergent individuals. Sixteen linear and angular measurements were used to assess both positions and inclinations of maxillary base, mandibular base, maxillary incisors, mandibular incisors, and inter-incisors relationship. Descriptive statistics were calculated and presented. The intra-class correlation coefficient (ICC) was applied for the reliability of measurements. Pearson correlation was used to present the degree of correlation between all variables. A *P* value of < 0.05 was considered significant.

**Results:**

There was a significant correlation between anteroposterior skeletal discrepancy and maxillary and mandibular dentoalveolar compensation (*P*<0.001). There was significant correlation between vertical skeletal discrepancy and maxillary and mandibular dentoalveolar compensation except for maxillary incisor position. Anterioposterior skeletal jaw position had greater significant effect on the dentoalveolar changes than the vertical skeletal jaw inclination did with variant degree.

**Conclusions:**

There may be an association between dentoalveolar changes and the skeletal anteroposterior and vertical positions, inclinations and intermaxillary relation.

** Key words:**Dentoalveolar compensation, anteroposterior, vertical, skeletal malocclusions, Cephalometry.

## Introduction

Malocclusion is abnormal variation from normal or ideal occlusion (1). This abnormality is a mainly result of perversion of normal growth and development (2). It might occurs in any of the three plan of space: anteroposterior, vertical and transverse. The deviations from normal can be presented clinically either in skeletal and/or dental form. Malocclusions of skeletal origin are more difficult to manage and to maintain, both manoeuvres become more challenging with increased skeletal discrepancy. The most common discrepancies in daily orthodontic loads are anteroposterior and vertical discrepancies (3).

Anterioposteriorly, the malocclusion presents in skeletal Class I, Class II and Class III malocclusion. Skeletal Class I is a normal sagittal relationship between maxillary and mandibular bases, although these bases might be in a normal or abnormal relation relative to the cranial base. The maxillary and mandibular relation is either exaggerated or reversed relative to normal relation in skeletal Class II and Class III, respectively (4).

Vertically, the malocclusion occurs in skeletal normodivergent, hypodivergent and hyperdivergent pattern. Skeletal normodivergent is a normal vertical relationship between maxillary and mandibular bases, although these bases might be in a normal or abnormal vertical relation relative to the cranial base. The maxillary and mandibular basal relation is either diverged or converged in skeletal hyperdivergent and hypodivergent patterns, respectively (5).

These skeletal variations from normal might be accompanied with changes in the dentoalveolar segment which is known as dentoalveolar compensation. Dentoalveolar compensation is ‘a system which can attain and maintain a normal relation with varying skeletal patterns’ (6). This compensation can be transversely in arch dimension to compensate for transvers skeletal discrepancy, vertically in dentoalveolar height to compensate for vertical skeletal discrepancy, and anterioposteriorly in the position and/or inclination of maxillary and mandibular incisors to compensate for anteroposterior skeletal discrepancy (2). All these compensations aim to camouflage for the skeletal disharmony to preserve the overall harmony and proportions of the dentofacial components. The factors responsible for dentoalveolar adaptation include: a normal eruptive system, surrounding soft tissue pressures and the influence by the neighbouring and opposing teeth during occlusion (7).

During orthodontic diagnosis and treatment planning, the orthodontists must measure the amount of skeletal discrepancies and the degree of dental compensation in the three plans as the treatment objective of any orthodontic cases may involve teeth movement and/or jaw movement either by growth modification or orthognathic surgery (8). In teeth movements approach, the treatment objectives are the necessary compensatory changes in tooth position relative to their basal bone (9). In jaw movement approach, the treatment objectives are to eliminate some of dentoalveolar compensation in an attempt to move the underlying skeletal bases to their normal position and relation in relation to each other and to the cranial base (10,11).

There are several linear and angular measurements used to evaluate the degree of skeletal discrepancies and dentoalveolar compensation (12-15). It is evident that angular measurements are less reliable in hyperdivergent face, so it is preferred that cephalometric analysis should base on both types of measurements (16-18). There are serval studies in orthodontic literature having the same aims (2,9,19-23), but comprehensive evaluation in anteroposterior and vertical in both position and inclination was not addressed yet. The aim of this retrospective study is to evaluate the position and inclination of maxillary and mandibular incisors in adults with different anteroposterior and vertical skeletal malocclusions.

## Material and Methods

This retrospective cross-sectional study was ethically approved by the Internal Review Board, College of Dentistry, Jazan University, Saudi Arabia. Lateral cephalometric images, demographic and clinical data of all patients who attended the orthodontic clinics between September 2011 and May 2017 were reviewed. Patients were included if they fulfilled the following inclusion criteria: 1) age above 17 years old, 2) no hereditary diseases or syndromes affected the craniofacial area, 3) no history of orthodontic treatment, 4) no major surgery in the craniofacial region. All patients signed an electronic informed consent before registration in the institute database; this consent includes their approval to use any patients’ data for research purposes.

The lateral cephalometry of patients fulfilled the inclusion criteria were digitally analysed. During imaging, the Frankfort horizontal was made parallel to the floor. All patients were instructed to bite in maximum intercuspation during cephalometric imaging to avoid any false vertical skeletal discrepancy.

The sample size was calculated using the G power software (Universität Düsseldorf, Düsseldorf, Germany). An alpha value of 0.05 and a power of 90% were considered. The calculation was based on a previous study conducted by Freudenthaler *et al.* (3) in which the mandibular incisors inclination to MP line was 94±10° for Class II and 82.5± 5.8° for Class III malocclusions, respectively and another study conducted by Modi *et al.* (19) in which the mandibular incisors position to NB line was 31.45±7.69mm for hyperdivergent face and 26.15 ±6.62mm for hypodivergent pattern, respectively. Power analysis indicated that 15 patients in each skeletal Class and 51 patients in each facial pattern were needed.

Two hundred seventy two patients fulfilled the selection criteria (134 males and 138 females). All patients were categorized anterioposteriorly based on angular and linear measurements; ANB angle (14) and A-B difference to Nasion vertical (mm) (13) into skeletal Class I, Class II and Class III malocclusion and vertically based on maxillo-mandibular plan angle (MMP) and Jarabak ratio (S-Go “mm”: N-Me “mm”) into normodivergent, hypodivergent and hyperdivergent individuals (15).

**Figure 1:**
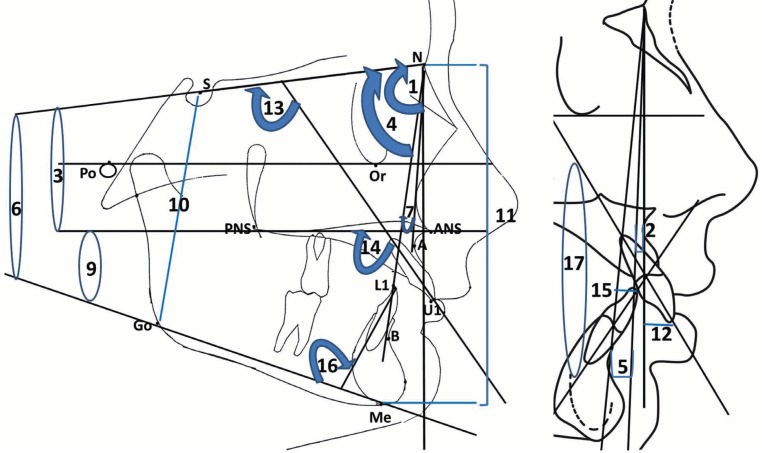
Skeletal and dentoalveolar measurements (1) SNA, (2) A-NV, (3) PP/SN, (4) SNB, (5) B-NV, (6) MP/SN, (7) ANB, (8) AB diff with NV, (9) MMP, (10) S-Go, (11) N-Me, (12) U1-NA, (13) U1/SN, (14) U1/PP, (15) L1-NB, (16) L1/MP, (15) U1-L1.

Lateral cephalometric skeletal and dentoalveolar measurements included linear and angular measurements as follows (Fig. 1):

(A) Skeletal Measurements

1- Maxillary base:

SNA: The angle between 3 point landmarks S, N and A points, determining the anteroposterior position of the maxilla relative to the cranial base.

A-NV: The linear distance measured between point A and Nasion vertical line, measuring the anteroposterior position of the maxilla relative to the Nasion vertical line.

PP/SN: The angle between Sella-Nasion (SN) and ANS-PNS (PP), determining the vertical position of the maxillary base relative to the cranial base.

2- Mandibular base:

SNB: The angle between 3 point landmarks S, N and B points, determining the anteroposterior position of the mandible relative to the cranial base.

B-NV: The linear distance measured between point B and Nasion vertical line, determining the anteroposterior position of the mandible relative to the Nasion vertical line.

MP/SN: The angle between Sella-Nasion (SN) and Go-Me (MP), determining the vertical position of the mandibular base relative to the cranial base.

3- Skeletal Maxillo-mandibular Relation:

ANB: The angle between 3 point landmarks A, N and B points, determining the anteroposterior jaw relation.

AB Diff–NV: The linear differences between A-NV and B- NV, determining the anteroposterior jaw relation.

MMP: The angle between the palatal plan and mandibular plan, determining the vertical jaw relation.

Jarabak ratio: It is the ratio of posterior to anterior facial height, determining the vertical facial proportion.

(B) Dentoalveolar Measurements

1- Maxillary Incisors:

U1-NA: The linear distance between Nasion-point A line and the most protruded point in the maxillary incisors.

U1/SN: The angle between the long axis of the most protruded maxillary incisor and the Sella-Nasion (SN) line.

U1/PP: The angle between the long axis of the most protruded maxillary incisor and the ANS-PNS (PP) line.

2- Mandibular Incisors:

L1-NB: The linear distance between Nasion-point B line and the most protruded point in the mandibular incisors.

L1/MP: The angle between the long axis of the most protruded mandibular incisor and the Go-Me (MP) line.

3- Inter-incisal angle (U1/L1):

The angle between the long axes of the most protruded maxillary and mandibular incisor.

Thirteen lateral cephalometric radiographs were selected randomly and measured independently by two examiners on two occasions at 2-week intervals to assure the reliability of readings. Data were inputted and analysed using Statistical Package for the Social Sciences (SPSS) software, Version 21 (Armonk, NY: IBM Corp.) for Windows. The intra-class correlation coefficient (ICC) was applied for the reliability of measurements. Descriptive statistics, including the mean and standard deviation for each variable, were calculated and presented. Pairwise comparisons by Class of malocclusion and vertical facial height were tested using independent t-test. A *P* value of < 0.05 was considered significant.

## Results

The sample comprised 272 patients; 134 were males (49.26%) and 138 were females (51.74%). Of the 272 patients included, 131 (48.16%), 83 (30.51%), and 58 (21.32%) presented as skeletal Class I, II and Class III, respectively. Of the 272 patients included, 54 (19.85%), 133 (48.89%), and 85 (31.25%) presented as hypodivergent, normodivergent and hyperdivergent pattern, respectively.

Intra- and inter-examiner reliabilities were high, with ICC values ranging between 0.825 and 0.990 for skeletal and dentoalveolar measurements. Descriptive statistics of the whole sample and each subgroup in anteroposterior and vertical skeletal and dentoalveolar measurements used in the study are presented in  Table 1 and  Table 2, respectively.

**Table 1:**
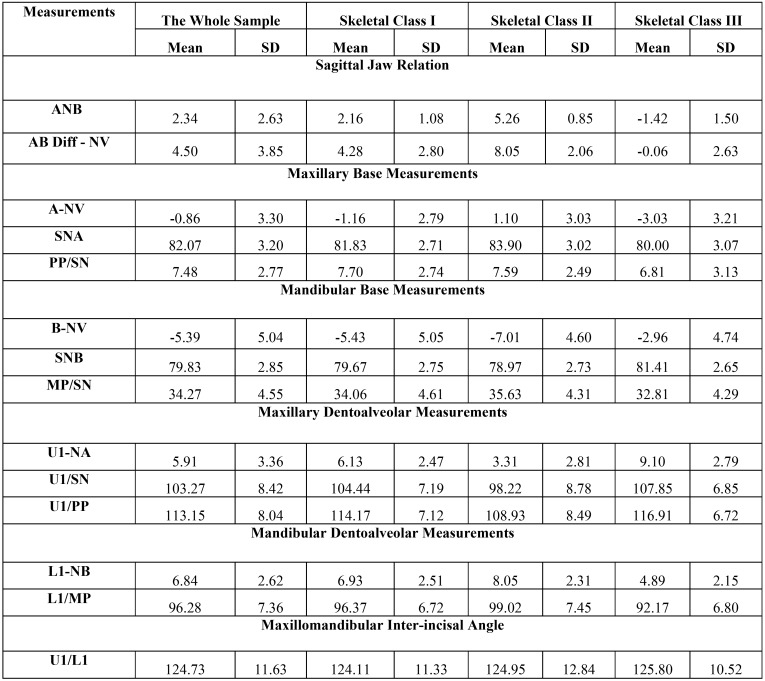
Descriptive statistics of anterioposterior skeletal and dentoalveolar measurements used in the study.

**Table 2:**
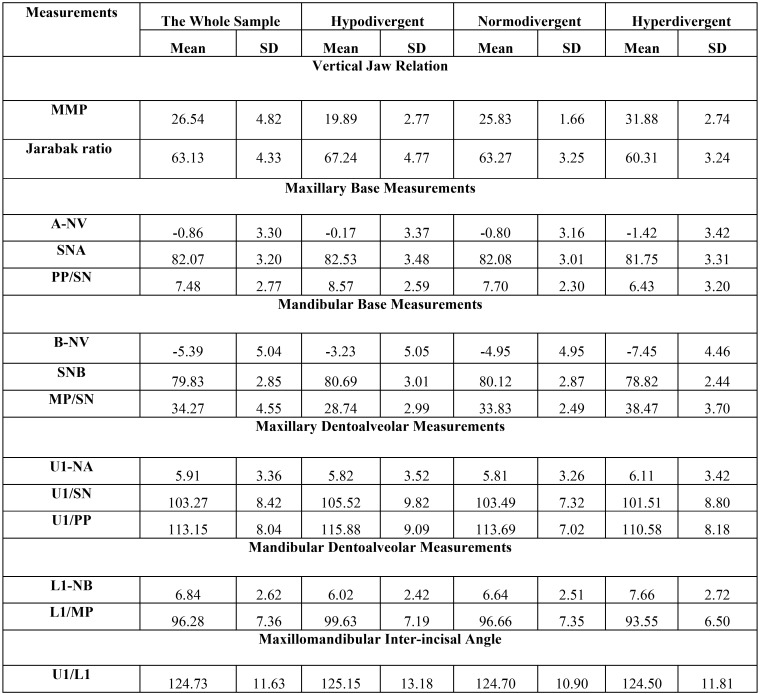
Descriptive statistics of vertical skeletal and dentoalveolar measurements used in the study.

The whole sample is more representative to evaluate this type of correlation. Regarding anterioposterior skeletal and dentoalveolar measurements, Pearson correlation coefficients showed significant (<0.001) negative correlation between sagittal discrepancy, ANB and maxillary incisors position (-0.676**), maxillary incisors inclination (-0.426**) and positive correlation with mandibular incisors position (0.461**) and inclination (0.368**). There was a significant positive correlation between maxillary base position (SNA) and mandibular incisors position (0.320**) and inclination (0.205**). The same result extend to the correlation between mandibular base position (SNB) and maxillary incisors position (0.255**) and inclination (0.495**). The correlations between skeletal bases and dentoalveolar parameters in each skeletal class were variant ( Table 3).

**Table 3:**
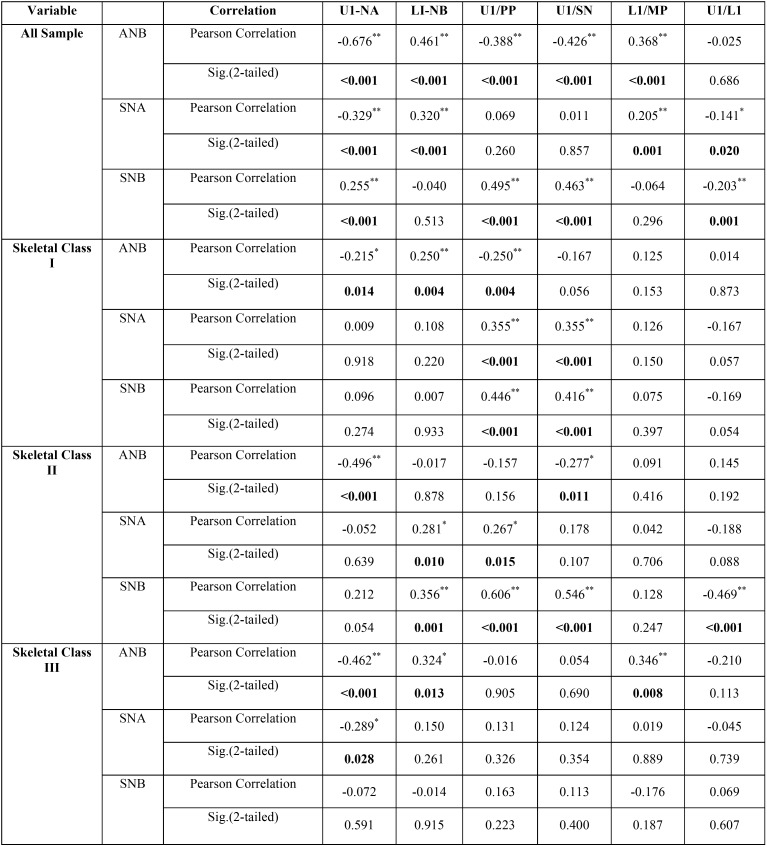
Pearson correlation coefficients between anterioposterior skeletal relation and dentoalveolar measurements [low correlation (˂<.31) medium (between 0.31 and 0.67) and high correlation (>0.7)].

For vertical skeletal and dentoalveolar measurements, Pearson correlation coefficients showed significant (<0.001) negative correlation between vertical discrepancy, MMP and maxillary (-0.219**) and mandibular incisors inclination (-0.310**), and positive correlation with mandibular incisors position (0.278**). The maxillary base inclination had minimal effect on the dentoalveolar parameters. The more significant correlation was between mandibular base inclination (MP/SN) and all dentoalveolar parameters except maxillary incisors position which was the least affected by the vertical

discrepancies. Mandibular base inclination had negative correlation with maxillary (-0.194**) and mandibular incisors inclination (-0.344**), and positive correlation with mandibular incisors position (0.279**). The correlations between vertical patterns and dentoalveolar parameters in each facial form are less variant than that for skeletal classes ( Table 4).

**Table 4:**
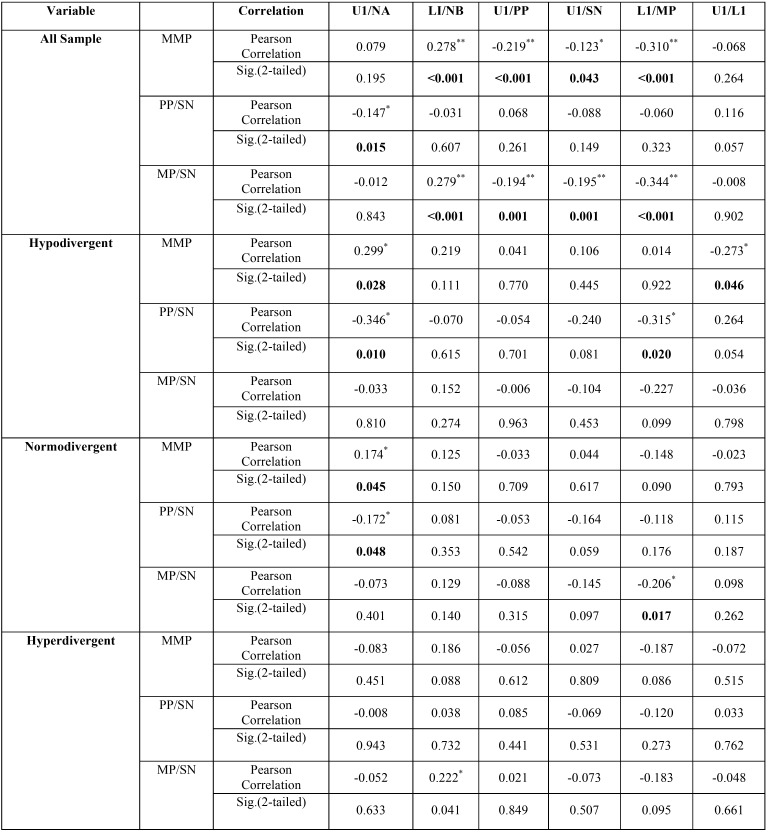
Pearson correlation coefficients between vertical skeletal relation and dentoalveolar measurements [low correlation (˂<0.31) medium (between 0.31 and 0.67) and high correlation (˂>0.7)].

## Discussion

Dentoalveolar compensation is a natural system for camouflaging skeletal discrepancies in three planes of space (6,23). During normal facial growth and development, there are two types of compensatory mechanisms, full compensatory occlusal development which enables normal relation despite some abnormalities in skeletal relationships, whereas, in contrast, insufficient compensatory guidance of tooth eruption can lead to future malocclusion (24).

Adult patients with a skeletal discrepancy can be treated with growth modification, orthodontic camouflage or orthognathic surgery, in which proper dentoalveolar compensation or decompensation is required for a successful treatment outcome (25-28). So, anterior dentoalveolar compensation is the main and the corner stone for successful orthodontic treatment either by orthodontic compensation (camouflage) or decompensation (skeletal base correction).

In this study the degree of compensation in different sagittal and vertical malocclusion were evaluated by correlation analysis. In sagittal discrepancy, there was an association between ANB (degree of anterioposterior discrepancy) and maxillary and mandibular incisors position and inclination, this association was negative with maxillary and positive with mandibular incisors. This indicated that with more increase in the overjet or sagittal discrepancy, the maxillary incisor becomes more retuded and retroclined while the mandibular incisors become more protruded and proclined to minimize the differences in basal malrelation. This was in accordance with other studies (9,21,22) In line with that Ceylan *et al.* (23) found positive correlation with maxillary incisor positions relative to NA line and no relation with mandibular ones. This difference mostly due the difference in sagittal measurement as they considered the overjet as a correlative item while our study based on skeletal correlative item.

Regarding the correlation between jaw bases and dentoalveolar parameters, there was a positive correlation between position of maxillary and mandibular base position with the mandibular and maxillary incisors position, respectively. This indicated that the dentoalveolar compensation is mostly affected by the discrepancy in the opposing rather than the holding jaw base. This confirms the concept of the camouflage by masking the abnormality. The correlation between the jaw base and the dentoalveolar parameters was not addressed in other studies except for Anwar and Fida (2) who correlated the chin position NP-Pog with lower incisors inclination and found unexplained results (positive correlation with L1/OP and L1/FH and no correlation with L1/SN and L1/MP).

Regarding the potential correlations between the different maxillo-mandibular vertical relationship (MMP) and the dentoalveolar parameters, the results showed no correlation with maxillary incisor position and significant negative correlation with maxillary and mandibular incisor inclinations and positive with mandibular incisors position. This indicates that with increased vertical jaw separation, both maxillary and mandibular incisors try to become more retroclined as retroclination always close the anterior open bite while proclination open the anterior deep bite. There was minimal effect of the maxillary base inclination on the dentoalveolar compensation and most of these effects were due to mandibular base inclination. This is exactly the biomechanical concept behind camouflaging anterior open or deep bite by retroclination or proclination of incisors, respectively (29).

There are several studies evaluated the dentoalveolar compensation in different vertical patterns (19,20,30) The correlation with maxillary and mandibular incisors position was not addressed in any previous study. For the negative correlation between maxillary and mandibular inclination with vertical patterns, Kuitert *et al.* (20) found the same findings in both short face and long face group while Modi *et al.* (19) found the same negative correlation with mandibular incisors inclination and no significant correlation with maxillary incisors inclination.

## Conclusions

From this cephalometric evaluation the following could be concluded:

1. There might be an association between dentoalveolar compensation and anterioposterior and vertical jaw relation.

2. Sagittal discrepancy has positive correlation with maxillary and mandibular incisors position and inclination.

3. Maxillary and mandibular bases have positive correlation with position and inclination of the opposing incisors.

4. Vertical pattern has almost negative correlation with maxillary and mandibular incisors.

5. Mandibular base inclination affected the degree of dentoalveolar compensation more than the maxillary base did.

However, there is still an urgent need for further three dimensional rather than two dimensional researches in this regard to confirm the evidence currently in hand.

## References

[1] Hassan R, Rahimah A (2007). Occlusion, malocclusion and method of measurements-an overview. Archives of orofacial sciences.

[2] Anwar N, Fida M (2009). Evaluation of dentoalveolar compensation in skeletal class II malocclusion in a Pakistani University Hospital setting. J Coll Physicians Surg Pak.

[3] Freudenthaler J, Celar A, Ritt C, Mitterocker P (2017). Geometric morphometrics of different malocclusions in lateral skull radiographs. J Orofac Orthop.

[4] Angle EH (1899). Classification of malocclusion. Dent Cosmos.

[5] Moyers RE, Riolo ML, Guire KE, Wainright RL, Bookstein FL (1980). Differential diagnosis of class II malocclusions. Part 1. Facial types associated with class II malocclusions. Am J Orthod.

[6] Solow B (1980). The dentoalveolar compensatory mechanism: background and clinical implications. Br J Orthod.

[7] Larson BE (2014). Orthodontic preparation for orthognathic surgery. Oral and Maxillofacial Surgery Clinics.

[8] Sarhan OA (1989). Rotational effects of SN on the dentoskeletal pattern within the range of normal. The Angle Orthodontist.

[9] Ishikawa H, Nakamura S, Iwasaki H, Kitazawa S, Tsukada H, Chu S (2000). Dentoalveolar compensation in negative overjet cases. The Angle Orthodontist.

[10] De Clerck HJ, Proffit WR (2015). Growth modification of the face: A current perspective with emphasis on Class III treatment. Am J Orthod Dentofacial Orthop.

[11] Naran S, Steinbacher DM, Taylor JA (2018). Current Concepts in Orthognathic Surgery. Plast Reconstr Surg.

[12] Steiner CC (1953). Cephalometrics for you and me. American journal of orthodontics and dentofacial orthopedics.

[13] McNamara Jr JA (1984). A method of cephalometric evaluation. American journal of orthodontics.

[14] Downs WB (1956). Analysis of the dentofacial profile. The Angle Orthodontist.

[15] Siriwat PP, Jarabak JR (1985). Malocclusion and facial morphology is there a relationship? An epidemiologic study. The Angle Orthodontist.

[16] Jacobson A (1976). Application of the" Wits" appraisal. American Journal of Orthodontics.

[17] Chang HP (1987). Assessment of anteroposterior jaw relationship. American Journal of Orthodontics and Dentofacial Orthopedics.

[18] Almaqrami B S, Alhammadi M S, Cao B (2018). Three dimensional reliability analyses of currently used methods for assessment of sagittal jaw discrepancy. Journal of clinical and experimental dentistry.

[19] Modi BN, Prakash A, Shetty S, Roy E (2013). Assessment of Dentoalveolar Compensation in Subjects with Vertical Skeletal Dysplasia: A Retrospective Cephalometric Study. Journal of Indian Orthodontic Society.

[20] Kuitert R, Beckmann S, van Loenen M, Tuinzing B, Zentner A (2006). Dentoalveolar compensation in subjects with vertical skeletal dysplasia. American journal of orthodontics and dentofacial orthopedics.

[21] Kim SJ, Kim KH, Yu HS, Baik HS (2014). Dentoalveolar compensation according to skeletal discrepancy and overjet in skeletal Class III patients. American Journal of Orthodontics and Dentofacial Orthopedics.

[22] Ishikawa H, Nakamura S, Iwasaki H, Kitazawa S, Tsukada H, Sato Y (1999). Dentoalveolar compensation related to variations in sagittal jaw relationships. The Angle Orthodontist.

[23] Ceylan I, Yavuz İ, Arslan F (2003). The effects of overjet on dentoalveolar compensation. The European Journal of Orthodontics.

[24] Bjo A, Skieller V (1972). Facial development and tooth eruption: An implant study at the age of puberty. American journal of orthodontics.

[25] Troy BA, Shanker S, Fields HW, Vig K, Johnston W (2009). Comparison of incisor inclination in patients with Class III malocclusion treated with orthognathic surgery or orthodontic camouflage. American Journal of Orthodontics and Dentofacial Orthopedics.

[26] Eslami S, Faber J, Fateh A, Sheikholaemmeh F, Grassia V, Jamilian A (2018). Treatment decision in adult patients with class III malocclusion: surgery versus orthodontics. Progress in orthodontics.

[27] Uribe F (2019). Orthodontic Considerations in Orthognathic Surgery. Aesthetic Orthognathic Surgery and Rhinoplasty.

[28] Elfeky HY, Fayed MS, Alhammadi MS, Soliman SAZ, El Boghdadi DM (2018). Three-dimensional skeletal, dentoalveolar and temporomandibular joint changes produced by Twin Block functional appliance. Journal of Orofacial Orthopedics/Fortschritte der Kieferorthopädie.

[29] Anwar N, Fida M (2009). Compensation for vertical dysplasia and its clinical application. The European Journal of Orthodontics.

[30] Buczko P, Szarmach I, Grycz M, Szarmach I (2015). Influence of vertical dimension on the degree of dentoalveolar compensation in patients with severe Class III malocclusion. Journal of Stomatology.

